# Crowd-sourced Ontology for Photoleukocoria: Identifying Common Internet Search Terms for a Potentially Important Pediatric Ophthalmic Sign

**DOI:** 10.1167/tvst.7.1.18

**Published:** 2018-02-15

**Authors:** Sandra E. Staffieri, Lisa S. Kearns, Paul G. Sanfilippo, Jamie E. Craig, David A. Mackey, Alex W. Hewitt

**Affiliations:** 1 Centre for Eye Research Australia, Royal Victorian Eye and Ear Hospital, Melbourne, VIC, Australia; 2 Ophthalmology, University of Melbourne, Department of Surgery, Melbourne, VIC, Australia; 3 Department of Ophthalmology, Flinders University, Flinders Medical Centre, Adelaide, SA, Australia; 4 Menzies Institute for Medical Research, School of Medicine, University of Tasmania, Hobart, TAS, Australia; 5 Lion's Eye Institute, Centre for Ophthalmology and Visual Sciences, University of Western Australia, Perth, WA, Australia

**Keywords:** retinoblastoma, leukocoria, information-seeking behavior

## Abstract

**Purpose:**

Leukocoria is the most common presenting sign for pediatric eye disease including retinoblastoma and cataract, with worse outcomes if diagnosis is delayed. We investigated whether individuals could identify leukocoria in photographs (photoleukocoria) and examined their subsequent Internet search behavior.

**Methods:**

Using a web-based questionnaire, in this cross-sectional study we invited adults aged over 18 years to view two photographs of a child with photoleukocoria, and then search the Internet to determine a possible diagnosis and action plan. The most commonly used search terms and websites accessed were recorded.

**Results:**

The questionnaire was completed by 1639 individuals. Facebook advertisement was the most effective recruitment strategy. The mean age of all respondents was 38.95 ± 14.59 years (range, 18–83), 94% were female, and 59.3% had children. An abnormality in the images presented was identified by 1613 (98.4%) participants. The most commonly used search terms were: “white,” “pupil,” “photo,” and “eye” reaching a variety of appropriate websites or links to print or social media articles.

**Conclusions:**

Different words or phrases were used to describe the same observation of photoleukocoria leading to a range of websites. Variations in the description of observed signs and search words influenced the sites reached, information obtained, and subsequent help-seeking intentions.

**Translational Relevance:**

Identifying the most commonly used search terms for photoleukocoria is an important step for search engine optimization. Being directed to the most appropriate websites informing of the significance of photoleukocoria and the appropriate actions to take could improve delays in diagnosis of important pediatric eye disease such as retinoblastoma or cataract.

## Introduction

The ‘red reflex' occurs when unobstructed visible light enters the eye through the pupil, and is reflected back from the vascular layer of the eye.^1^ While a ‘red eye' may commonly appear in photographs, particularly when a flash is used, a white reflex or pupil (leukocoria) can be a sign of significant pediatric eye disease. Testing the red (Brückner) reflex has long been used as a screening tool in newborns and for postnatal eye examinations to detect the presence of any intraocular pathology that could compromise normal vision development,^2–4^ as well as detecting the presence of strabismus or anisometropia.^5^

Leukocoria can herald the presence of congenital cataract or retinoblastoma in children, and less frequently Coats' disease, retinal detachment, or persistent fetal vasculature.^6,7^ Early recognition of this clinical sign and prompt differential diagnosis, as well as treatment of its underlying cause, affords the child the best possible visual outcome.^8–11^ In the case of retinoblastoma, the most common pediatric intraocular neoplasm,^12^ early diagnosis can be life saving. Indeed, published reports of delayed diagnosis of retinoblastoma indicate that although parents are most likely the first to observe and report this finding, they are generally unaware that it is a sign of this potentially fatal disease.^11,13^

As the most common presenting sign for retinoblastoma^14–16^ leukocoria is often noticed by a child's caregiver in photographs (photoleukocoria) or simply by looking at the child.^17^ It may only be observed intermittently at different angles or lighting conditions, adding to the parents' delay in seeking medical advice.^11,18^ Leukocoria, however, can also result as an artefact of lighting or photographic angle—where light bounces off the optic nerve head or due to the type of camera used.^19^ In developed countries, more than 50% of retinoblastoma cases will present with leukocoria.^16,20,21^ In resource-poor settings, this statistic can reach as high as 80%^22^ or be as low as 30% when children are more likely to present with advanced, extraocular disease as the initial leukocoria has been missed or ignored.^23^ Educating caregivers about the importance of recognizing leukocoria early has been shown to significantly improve survival of children with retinoblastoma.^24^

The explosion of ready access to health information on the Internet is well recognized, and increasing at a rapid rate. The Pew Internet and American Life Project found that almost four of five individuals using the Internet to seek health-related information did so via a search engine.^25^ Further analysis confirmed that 28% of respondents indicated the information found on the Internet influenced their help-seeking behavior.^26^ eHealth literacy is defined as an individual seeking health-related information, being able to navigate the World Wide Web reliably and being directed to accurate, useful information to support their help-seeking decisions or behavior.^27^ It has also been demonstrated that eHealth illiteracy can result in delayed or self-treatment with the potential for poor outcomes.^28^ To ensure timely diagnosis and appropriate treatment, it is vital to develop credible and helpful information resources that incorporate the search terms people are most likely to use. The value and importance of accessible ophthalmic information on the Internet has been recognized with studies examining readability^29^ and quality of information in ophthalmology.^30^ Despite this, to date no published studies have investigated the search terms that might be used to initiate seeking health information for specific signs or symptoms of eye disease. However, developing the ontological relationship between the search words used and the websites reached must also be considered and it is even more imperative that this relationship be determined by the end-user. Crowd sourced ontology is an emerging technique for exploring and developing algorithms for biomedical ontology and its importance is paramount as more users seek health information on the World Wide Web.^31^

We investigated whether individuals from the general community could identify leukocoria in photographs (photoleukocoria) and if observed, how they used the Internet to seek further information. The aim of this project was to identify the most frequent free text search words that adults might use to seek further information using Internet search engines and to explore how this information would influence their help-seeking intentions.

## Methods

**Figure 1:**
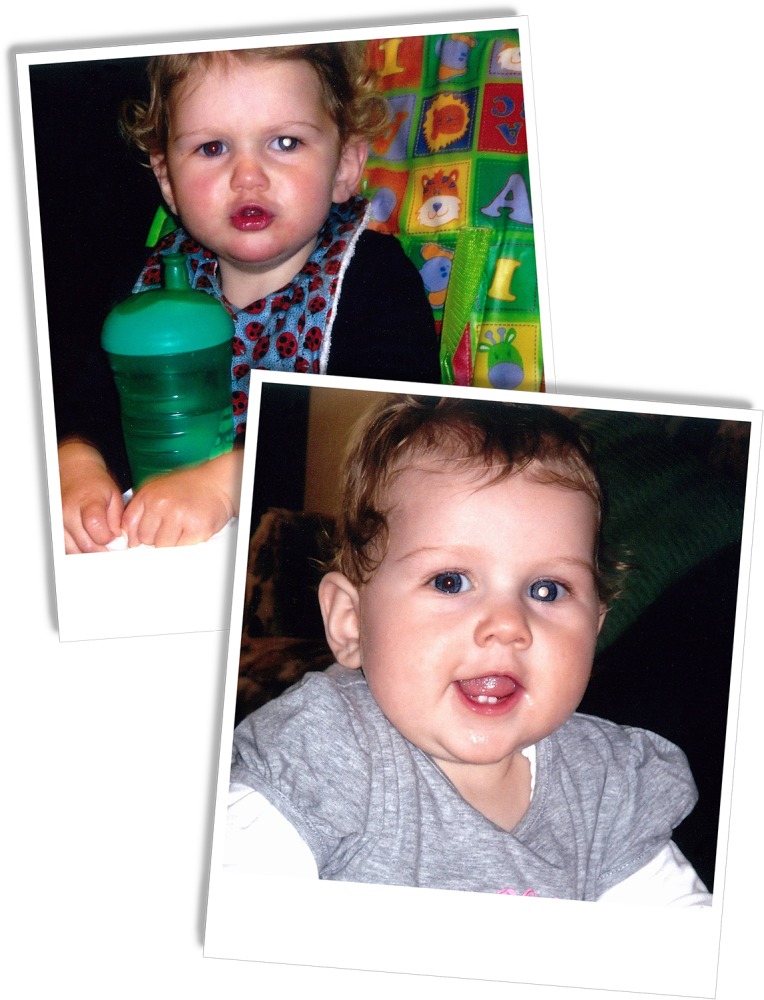
Images used in the online survey. Participants were informed that they had taken these photographs of a family member on separate occasions and then were asked to search the Internet for information about their observations.

A focus group of five parents naïve to the potential significance of leukocoria were recruited from a Community Health Centre. With written, parental permission for the use of their child's images, we showed two photographic prompts of a child with photoleukocoria to the focus group (Fig. 1). Draft paper survey questions were constructed to explore the ability of adults to recognize the photoleukocoria, their level of concern, and their hypothetical help-seeking intention. The survey questions were iteratively modified for content and comprehension in response to the focus group feedback until saturation was reached.

The paper survey was then developed as a web-based survey: www.checkthispicture.com for the collection of data for this project (Supplementary Methods S1). The study was approved by the Human Research Ethics Committee of the Royal Victorian Eye and Ear Hospital (HREC #13/1113HS) and adhered to the tenets of the Declaration of Helsinki.

In this cross-sectional study, adults (≥18 years) were invited to complete the survey via posters and newsletters at Victorian community centers and libraries (Supplementary Methods S2). Due to a slower than anticipated response rate, a paid advertisement on social media (Facebook, Menlo Park, CA) inviting participation in the online survey was commissioned (Supplementary Methods S3). The advertisement was electronically restricted to Facebook users over the age of 18 years and no financial or other rewards were offered to encourage participation. Information about the study was provided, with consent implied when the participant elected to complete the online survey. Basic demographic data were collected including: age, sex, geographic location, parental status, and healthcare background.

Participants were shown the same photographic prompts used in the focus groups (Fig. 1) and asked to indicate their concern and where they would go for advice. They were then invited to record their observations and search the Internet to ascertain more information. If the Internet search was conducted in the browser provided, the search words used and the resulting websites visited were recorded by the database. Following their Internet search, participants were required to suggest a possible diagnosis and indicate their help-seeking intention based on their findings. Data were collected and downloaded for analysis. Participants' suggested diagnoses were coded according to the following schema: 1 = retinoblastoma; 2 = leukocoria/any other cause of leukocoria (i.e., cataract/Coats' disease); 3 = any type of other eye disease; 4 = any disease; 5 = unknown/did not respond; 6 = camera/photography; 7 = nothing wrong.

The primary outcome of this study was the identification of the most common search words participants used to seek information on the Internet when shown a photographic prompt of photoleukocoria. Secondary outcomes included the sites or webpages reached using these search words; the participants' ability to correctly consider photoleukocoria as a significant problem; participants' sources of advice pre- and post Internet search; and their help-seeking intentions following the knowledge gleaned from their search. Help-seeking intentions were classified as: ‘appropriate'—to seek professional advice within 1 day or week and ‘inappropriate'—to seek professional advice within 1 month, 1 year, or no response.

To capitalize on the opportunity for community education, at the completion of the survey participants were provided with a link leading to important information about leukocoria.

All statistical analyses were performed in R (version 3.3.2; The R Project for Statistical Computing, Vienna, Austria), and data are presented as the mean ± standard deviation unless otherwise indicated.

## Results

### Cohort Demographics

The demographic characteristics of the participants are shown in Table 1. A total of 1639 adults, of whom 94% (*n* = 1541) were female, participated in the survey. The mean age for the total cohort was 38.95 ± 14.59 (range, 18–83) years. Despite the disproportionate representation of females to males, their mean age in years was similar and not statistically significant.

**Table 1:**
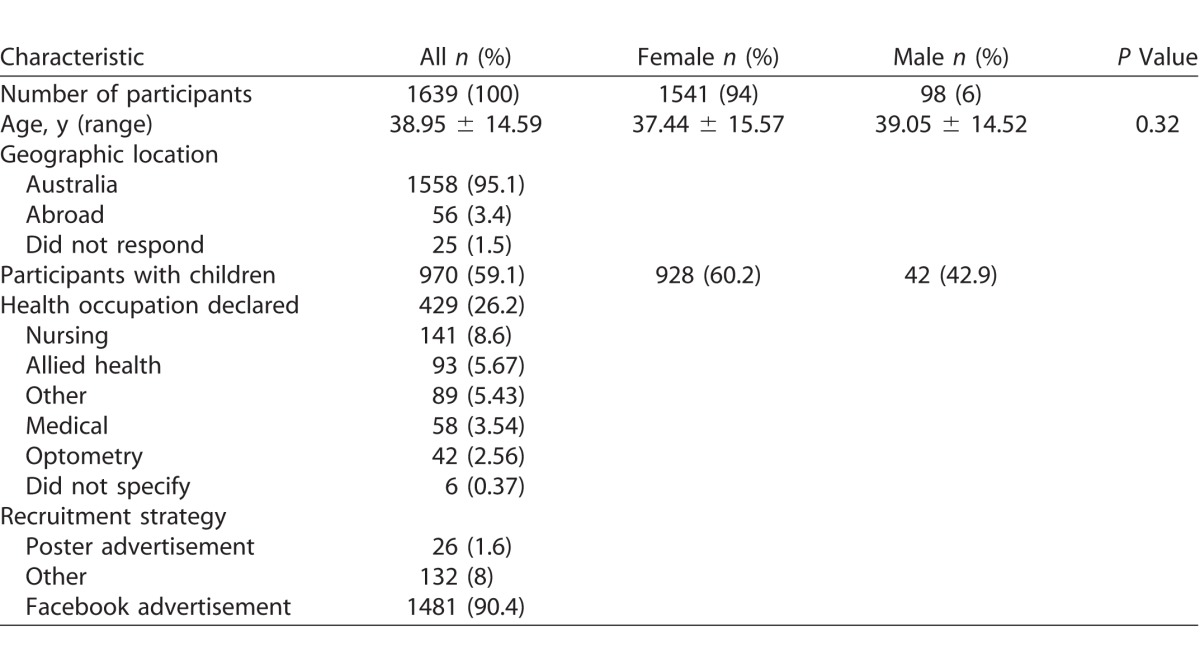
Participant Characteristics and Demographics

A total of 1558 (95.1%) participants resided in Australia and 970 (59%) reported being parents. While 429 (26.2%) declared a background in health care, nursing was the most commonly cited profession (141/429; 32.9%). Facebook was the most effective recruitment strategy with 1481/1639 (90.4%) participants recruited through this medium.

### Most Commonly Used Search Terms for Photoleukocoria

Of all participants, 816 (49.8%) conducted a web search in the field provided, enabling their search words and subsequent websites reached to be recorded for analysis. These 816 participants conducted a total of 1111 searches, accessing 310 different web pages across 170 websites.

As shown in Table 2, the 10 most commonly used search words were the same for all sites reached as those for reaching the top 10 sites as shown in Table 3.

**Table 2:**
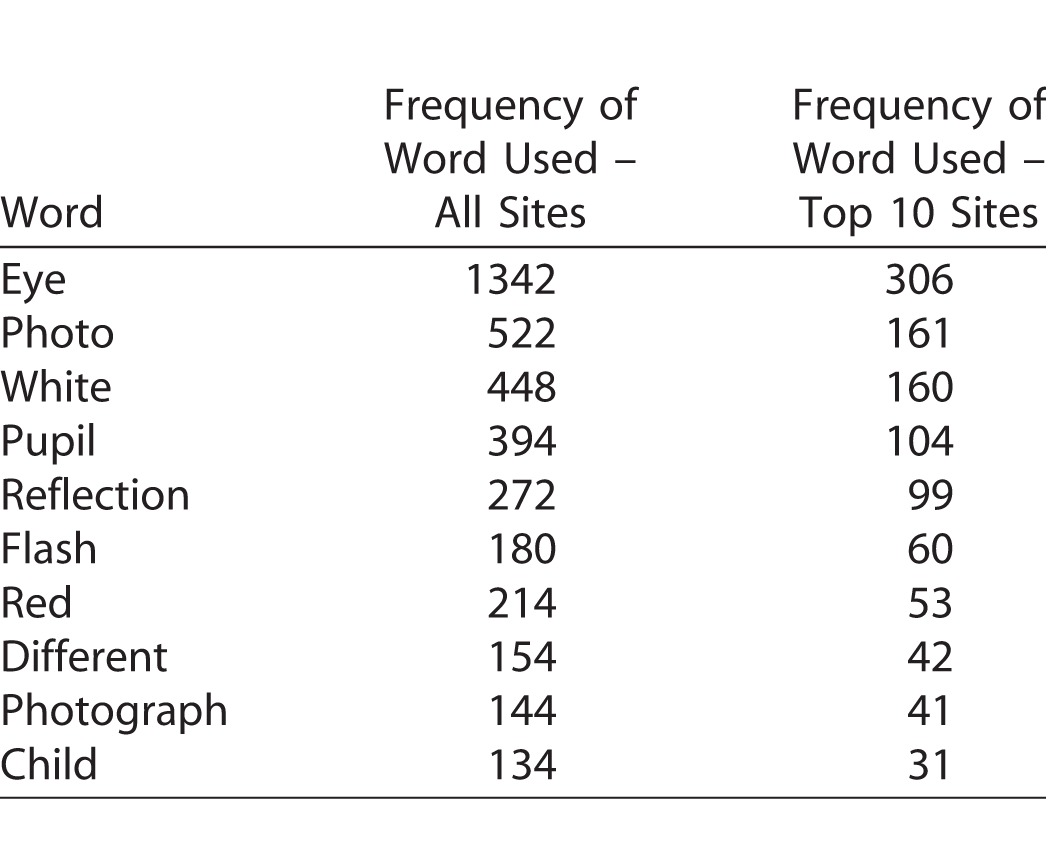
Top 10 Most Frequently Used Search Terms to Reach All Sites and the Top 10 Most Frequently Reached Sites as Shown in Table 3

**Table 3:**
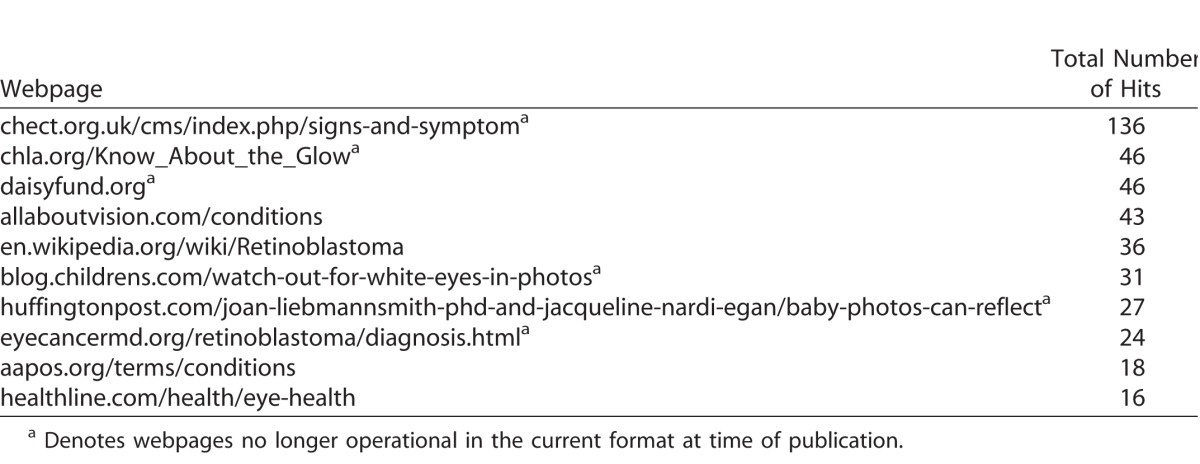
Top 10 Websites Viewed by Participants During Web Search

### Most Frequently Reached Websites

The web-based survey was designed to capture and store responses, including search terms used and websites or landing pages visited. The top 10 websites reached following participant web searches are shown in Table 3. Notably, the top and third top sites are retinoblastoma-specific charitable organizations, while the second top-site is a hospital-based retinoblastoma-specific webpage.

Web-search behavior varied from one to nine searches, (mean of 1.36 ± 0.94). Single words were not used to describe the observations in the photographs, unless the word used was an actual diagnosis, such as cataract, glaucoma, or retinoblastoma. Instead, a number of words or phrases were used in combination (e.g. “white pupil in photo” or “camera flash in one eye only”) to search for information on the Internet.

### Recognizing Photoleukocoria as a Problem

Of the total participating cohort, 1613 (98.4%) noted a problem in the photographs (Fig. 1); however, only 1394 (85%) were concerned by this observation. Following their Internet search, either in the field provided or another tab, 747/1639 (45.6%) participants correctly identified retinoblastoma as the most likely diagnosis. A further 244 (15.1%) concluded other causes of leukocoria, such as Coats' disease or cataract, as the most likely cause of the photoleukocoria. However, despite their searches, 48/1639 (2.9%) participants still considered the observed abnormality was due to a photographic artefact and 10/1639 (0.6%) did not notice any problem in the photographs at all.

### Sources of Advice Pre- and Post Internet Search

**Figure 2:**
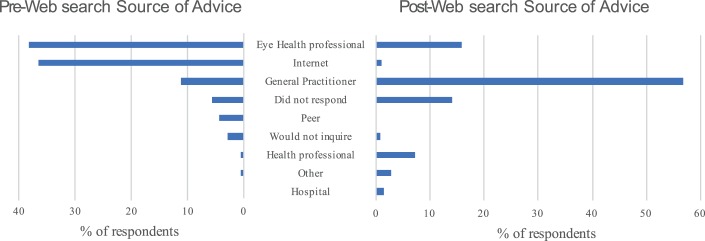
Breakdown of provider sought for advice before or after the participant's Internet search.

A significant shift in the source of advice was observed before and after participants' Internet searches. The Internet or an eye health professional was the preferred source of advice prior to their search—598/1639 (36.5%) and 626/1639 (38.2%), respectively. However, following their searches, there was a marked shift to seeking advice from a general practitioner from only 185/1639 (11.3%) to 931/1639 (56.8%) (Fig. 2).

### Help-Seeking Intentions Following Web Searches

The demographic profile of participants' help-seeking intentions according to their web search is shown in Table 4. There was no significant difference in help-seeking intention for sex; however, a statistically significant difference was found for older participants, participants with children, or participants who declared a background in healthcare.

**Table 4:**
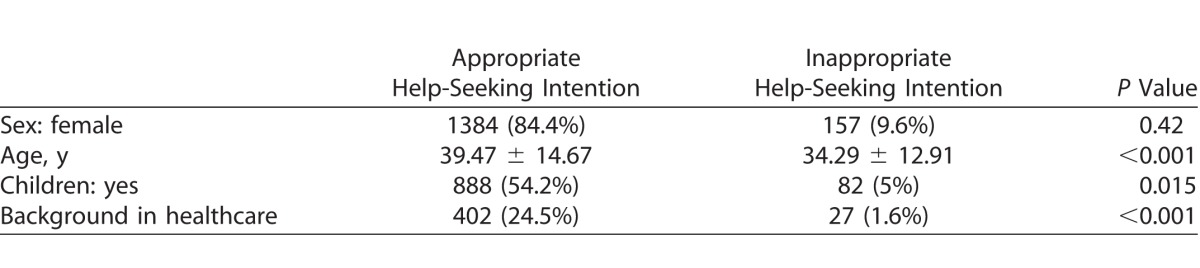
Comparison of Demographic Profiles Between Participants With Appropriate or Inappropriate Help-Seeking Intentions (n = 1639)

The same top three search terms, “eye,” “photo,” and “white,” as shown in Table 2, were used by participants in both the appropriate and inappropriate help-seeking intention groups. Moreover, despite reaching a retinoblastoma-relevant site or page, 52 (6.4%) participants still indicated an inappropriate help-seeking intention despite reaching the same informative webpages 27 times, which included those listed in the top 10 pages or sites reached in Table 3.

## Discussion

This study identified the most commonly used search terms for photoleukocoria and the top websites reached by an adult, largely nonmedical, cohort. Most participants obtained sufficient information to suggest an appropriate help-seeking intention and determine the best source of advice. Pehora and co-workers^32^ suggest reliable, readily accessible web-based health formation will continue as a key consideration for healthcare providers, public health officers, and web content developers. Optimization of relevant websites with the search terms we identified could direct caregivers who observe photoleukocoria to seek advice sooner, possibly leading to earlier treatment and better outcomes particularly for children with retinoblastoma. The broad spectrum of commonly used search terms and phrases characterized in this study should be adopted by the known organizations and web-developers providing information on pediatric eye health. In particular the phrases searched by people with an inappropriate help-seeking intention should also be incorporated into the search engine optimization of these web-based patient resources.

In common with other researchers we found that Facebook was the most effective recruitment method for this study.^33,34^ It is possible that participants who were already online (Facebook) and received the invitation to participate in the survey found it easier to do so in real time versus those who read about the study on a flier and then had to remember to log into the web-based survey at a later time.

Consistent with the literature,^35–37^ we also had more female participants. While it could be hypothesized that the photograph of a child in the invitation to participate in the project may have attracted more mothers as the most common primary caregiver, only 60.2% of all females in this cohort had children.

The moderately high rate of healthcare workers in this study sample was unexpected and may have biased the outcome, as it is presumed their knowledge and e-health literacy skills could be greater than the general population. While it is not known whether the healthcare workers recognized the signs of retinoblastoma in the photographic prompt before their web search, it is worth noting that the majority were able to decide on the appropriate help-seeking intention. Future studies could explore this further.

Not all the searches were undertaken in the field provided, limiting the capture of the Internet search behavior of all participants. It is presumed however that participants whose search strategy could not be tracked also underwent a similar iterative process as has been described in qualitative analyses of online health information–seeking behavior.^38^ Interestingly, the most commonly used search terms of participants who determined the appropriate help-seeking intention as compared with those who did not were similar, as were the websites reached.

It is not clear why participants whose searches could be tracked disregarded the photoleukocoria as an artefact or were happy with an inappropriate help-seeking intention despite reading information regarding the potential cause of leukocoria. It is possible that with the frequent use of smartphones for photographs and the resulting increased observation of leukocoria as an artefact, the public may have become desensitized to its appearance and less likely to be concerned when it is observed. Additionally, leukocoria seen in dim lighting or ‘tapetum lucidum' is a commonly observed phenomenon in animals,^39^ and this may further desensitize the public to this important sign. Moreover, the use of technology to remove ‘red eye' in photographs delaying the diagnosis of retinoblastoma has been reported in the literature,^40^ suggesting abnormal looking pupils may be considered less of a sign of potential eye disease and more an inconvenience. Furthermore, the terminology used by the participants to describe their observations in their Internet search may have positively or negatively impacted on the resulting landing page reached, and their subsequent help-seeking intention.

In our study, the majority of websites reached were hospital or organization based. With the unregulated availability of health information on the Internet, credibility of such information is brought into question. Indeed, studies have identified trustworthiness of web-based information as important to the reader and that information found on hospital or organization based websites as the most trusted source.^32^ However, while information written by non–health professionals was more likely to be recognized as less reliable, articles in the media recounting personal stories of a diagnosis of retinoblastoma in our study were also reached, providing information regarding the potential significance of photoleukocoria. It is worth noting, however, that while media articles can be helpful, their lifespan on the Internet may be limited as they are often archived relatively quickly. Many sites reached by the participants are no longer active in their current state and thus highlight the importance of sustainable websites with relevant information.

The Question-Behaviour Effect is the phenomenon, whereby an individual, simply being asked a question, changes their behavior.^41^ Thus, we cannot know whether the participants would have noted the abnormal findings in the photographs if they were not prompted to do so. Indeed, Abdolvahabi's^19^ retrospective review of a vast bank of photographs of nine children with a subsequent diagnosis of retinoblastoma demonstrated the presence of photoleukocoria well before the parent observed the sign and was prompted to seek attention.

As previously reported in the literature,^42^ the Internet was the most commonly cited source of health information for this cohort. Nonetheless, it was noteworthy that following participants' Internet searches, help-seeking intentions changed from an eye care provider to a general practitioner. We are unclear as to the underlying reason for this, though postulate it could reflect the advice provided at the websites visited. It is possible participants responded to the likely suggestion at these sites to seek further advice from their primary health care provider or general practitioner.

There are several limitations in our study. Although we collected sex, parental status, and occupation, educational attainment was not recorded. This may have provided further insight into the demographic of Internet users and their search behaviors for health information. In addition, while participants' source of health information prior to their web search was recorded, the time period in which they would seek information or advice was not. This could have provided further evidence that the information gleaned from their web search had some impact on their help-seeking intentions, not just the source of advice.

Given the search words and resulting sites could be recorded for only 49.8% (816/1639) participants, it is possible, that some participants may have undertaken their web search in another browser. Similarly, participant Internet Protocol addresses were not recorded; however, given the majority of participants responded to the random appearance of a Facebook advertisement, it is unlikely that participants completed the survey more than once.

Future qualitative research could expand on this work to better understand an individual's process of searching the Internet to contribute to the development of useful and informative websites for health information. Furthermore, this study has only considered search words for the English language. Identifying the search terms for the observation of photoleukocoria in other languages could further expand the benefits of optimizing search words to lead to useful, reliable, and informative websites.

With rapid advances in the digital age, there is an increasing reliance on the Internet as an initial source of medical information. Identifying the most commonly used search terms for photoleukocoria is an important step to ontology development to optimize reliable sites accessed with useful information and assist the individual to make an informed and appropriate choice for seeking further medical advice. Variations in an individual's description of observed signs and search words used to seek information could influence the site reached, the information obtained, and their subsequent help-seeking intention.

## Supplementary Material

Supplement 1

Supplement 2

Supplement 3
