# Supplementary material for: Crowd-sourced Ontology for Photoleukocoria: Identifying Common Internet Search Terms for a Potentially Important Pediatric Ophthalmic Sign
Source: Transl Vis Sci Technol. 2018 Feb 15;7(1):18. doi: 10.1167/tvst.7.1.18 (PMC5815559; doi:10.1167/tvst.7.1.18)
Supplement: Supplement 1 [file tvst-07-01-08_s01.pdf]

When a picture paints more than a thousand words...

The purpose of this website is to investigate people’s online search behaviour for medical information.

You will be shown two photographs and asked to identify any noticeable problem, and search for a possible diagnosis.

Please take as much time as you require. Most people spend about 10 minutes.

Your identity is not required; however, you should include your email address if you would like to receive the results of this project.

Human Research and Ethics Approval has been granted to conduct this research project (RVEEH HREC: 13/1113HS). The researchers are obliged to abide by the regulations of the approval.

Scenario:

You have recently taken these photographs of a family member on separate occasions.

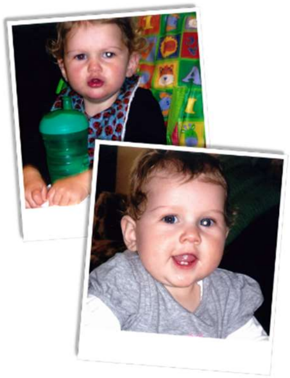

Task 1.

Do you notice anything about these photographs?

If you had taken these photographs would you be concerned?

How would you initially find out more information about what you notice?

You decide to search the internet...

Task 2

In the field below please search to find out what you think could be wrong with their eye. Click the next button when you think you know what it could be and what you should do about it. Please click on at least one search result.

Please begin typing your search query.

Next

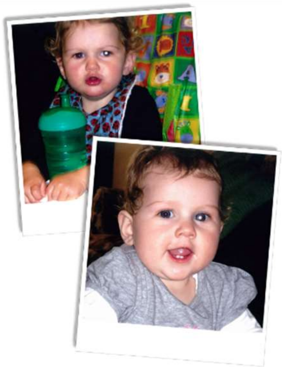

Task 3.

What do you think the most likely diagnosis is?

Next

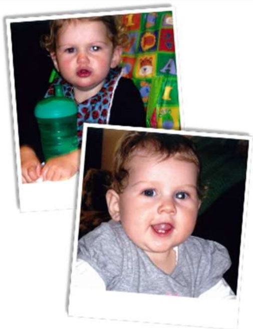

Task 4.

Could this be anything else?

Next

Task 5.

Would you do anything about this?

Next

Thank you for participating...

To receive a copy of the results of this survey, complete your email address:

Email Address:

Find out more? *The participant could select this button and the following information appeared:*

That glint should make you think...

"Leukocoria or white pupil reflex"

Often leukocoria is seen by the parent in family photographs or with the naked eye. Delay in diagnosis for retinoblastoma is common and can result in further advancement of the disease with adverse consequences. All published reports on delayed diagnosis of retinoblastoma recommend improved education for health practitioners and the general community alike of the potentially sinister sign of leukocoria. Such education programs have not been forthcoming, except for sporadic cases reported in the newspapers and magazines. Earlier diagnosis could save the child's vision, eye or their life.

This research project aims to identify the free text search words people might use to seek further information on photographic findings using online search engines. By determining the most commonly used free text search words, recommendations and changes can be made to ensure prompt access and direction via the internet to appropriate, reliable information regarding retinoblastoma and cataract, and the importance of seeking urgent referral to a medical practitioner. This better access may in turn lead to reduced delays in diagnosis and improved outcomes for the affected individual.

This work was supported by an Australian National Health and Medical Research Council Centre of Research Excellence in Translation of Genetic Eye Research.

- A 'white' pupil seen in photographs could indicate any of the following:
    - Reflection off the optic nerve – photographic artefact
    - Retinoblastoma
    - Cataract
    - Or other less common eye conditions.
- If you have taken a photograph like this, you should seek URGENT medical attention to confirm the diagnosis.
- Retinoblastoma (Rb) – malignant eye tumour
    - Can occur in one or both eyes
    - Can be present at birth, or develop in the first 5 years of life

    - Can be hereditary – if there is a history of Rb in your family you MUST seek further advice from an ophthalmologist (eye specialist) or genetic counsellor
    - Earlier detection can save the child's vision, eye or life
    - Untreated, Rb can be fatal
    - To find out more see www.daisyfund.org; www.chect.org.uk
  - Congenital Cataract – clouding of the lens in the eye

    - Can occur in one or both eyes
    - Can be present at birth or develop during childhood, particularly after severe eye injury
    - Can be hereditary
    - Prompt diagnosis and early treatment is important to achieve best vision
    - To find out more see http://kidshealth.schn.health.nsw.gov.au/fact-sheets/congenital-cataracts

- Contact the researchers...
    - Email: info@.checkthispicture.com

Translation Of Genetic Eye Research

National Health & Medical Research Council

Centre of Research Excellence

Sandra Staffieri, Lisa Kearns and Alex Hewitt
Clinical Genetics Unit, Centre for Eye Research Australia,Level 1, 32 Gisborne Street
East Melbourne, VIC 3002

Centre for Eye Research Australia

David Mackey – Lions Eye Institute, 2 Verdun Street, Nedlands, WA 6009

LIONS EYE INSTITUTE

Jamie Craig Department of Ophthalmology, Flinders University, Bedford Park, SA 5042

Flinders UNIVERSITY
