# Supplementary material for: Crowd-sourced Ontology for Photoleukocoria: Identifying Common Internet Search Terms for a Potentially Important Pediatric Ophthalmic Sign
Source: Transl Vis Sci Technol. 2018 Feb 15;7(1):18. doi: 10.1167/tvst.7.1.18 (PMC5815559; doi:10.1167/tvst.7.1.18)
Supplement: Supplement 2 [file tvst-07-01-08_s02.pdf]

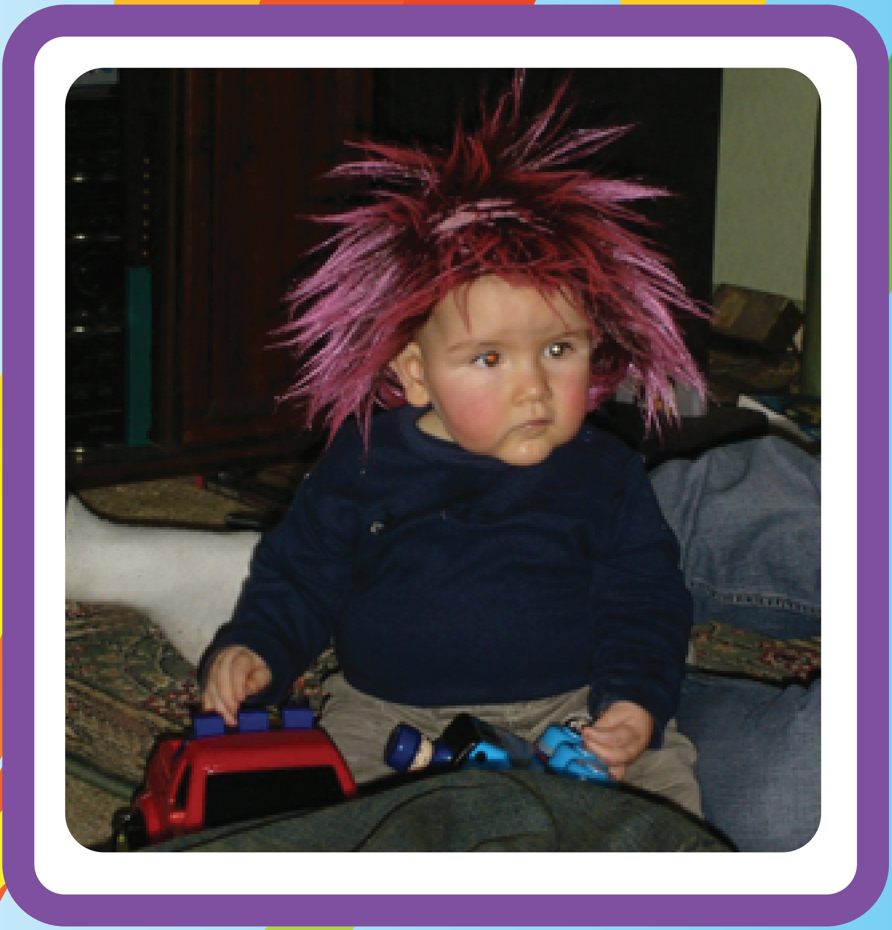

Log on to participate!
only takes 15 mins.

Every picture tells a story......

www.checkthispicture.com
